# Genome-wide alternative splicing landscapes modulated by biotrophic sugarcane smut pathogen

**DOI:** 10.1038/s41598-019-45184-1

**Published:** 2019-06-20

**Authors:** Renesh Bedre, Sonia Irigoyen, Patricia D. C. Schaker, Claudia B. Monteiro-Vitorello, Jorge A. Da Silva, Kranthi K. Mandadi

**Affiliations:** 1 Texas A&M AgriLife Research & Extension Center, Texas A&M University, Weslaco, TX USA; 2 Departamento de Genética, Universidade de São Paulo, Escola Superior de Agricultura “Luiz de Queiroz,” Piracicaba, São Paulo, Brazil; 3 Department of Soil & Crop Sciences, Texas A&M University, College Station, TX USA; 4 Department of Plant Pathology & Microbiology, Texas A&M University, College Station, TX USA; 5 Present Address: Universidade Tecnológica Federal do Paraná, Toledo, Paraná Brazil

**Keywords:** Biotic, Plant molecular biology

## Abstract

Alternative splicing (AS) promotes transcriptome and proteome diversity during growth, development, and stress responses in eukaryotes. Genome-wide studies of AS in sugarcane (*Saccharum* spp.) are lacking, mainly due to the absence of a high-quality sequenced reference genome, sugarcane’s large, complex genome, and the variable chromosome numbers and polyploidy of sugarcane cultivars. Here, we analyzed changes in the sugarcane isoform-level transcriptome and AS landscape during infection with the smut fungus (*Sporisorium scitamineum*) using a hybrid approach involving *Sorghum bicolor* reference-based and Trinity *de novo* mapping tools. In total, this analysis detected 16,039 and 15,379 transcripts (≥2 FPKM) at 5 and 200 days after infection, respectively. A conservative estimate of isoform-level expression suggested that approximately 5,000 (14%) sugarcane genes undergo AS. Differential expression analysis of the alternatively spliced genes in healthy and smut-infected sugarcane revealed 896 AS events modulated at different stages of infection. Gene family and gene ontology functional enrichment analysis of the differentially spliced genes revealed overrepresentation of functional categories related to the cell wall, defense, and redox homeostasis pathways. Our study provides novel insight into the AS landscape of sugarcane during smut disease interactions.

## Introduction

Sugarcane (*Saccharum* spp., Poaceae family) is a high-value C_4_ grass with a global estimated harvest yield of ~1.89 billion tons in 2016^1^, contributing to ~75% of sugar and ~60% of ethanol production worldwide^2,3^. The biotrophic fungal pathogen *Sporisorium scitamineum* (Syd.) (previously known as *Ustilago scitaminea*^4^; causes wind- and soil-borne smut disease. Smut symptoms are characterized by powdery masses of teliospores, which form long black or gray whip-like structures emerging from the primary meristem of the sugarcane plant^4–7^. These whip-like structures contain a mixture of plant and pathogen cells with millions of teliospores. The teliospores can spread throughout a field, rapidly advancing the disease to new areas^4,5,7^. Smut is found worldwide and causes serious damage to sugarcane yield, juice quality, culms, and sucrose content^5–8^. Losses can range from 30–100% and, in extreme cases, can cause the demise of local varieties^2,4,7,9^. The severity of damage depends mostly on the smut race, sugarcane genotype, and neighboring environmental conditions^7^.

Development of smut-resistant sugarcane varieties is an arduous task due to 1) the complex sugarcane–smut pathosystem, 2) the many genes controlling resistance, which acts as a quantitative trait, and 3) the current poor understanding of gene-for-gene resistance^7,8^. Furthermore, considering annual increases in prevalence of sugarcane smut disease and lack of control strategies, development of smut-resistant sugarcane varieties has emerged as a key priority. Developing such varieties by manipulating the host system likely represents a safe, effective, economical, and environmentally friendly way of controlling smut disease^2,7,8,10–13^. Therefore, understanding sugarcane genetic responses to smut could provide key information for developing smut-resistant varieties.

We, and others, have reported genetic- and genome-level studies of sugarcane–smut interactions^2,5,7,10,12,14^. For instance^10^, studied the comparative response of reactive oxygen species (ROS) metabolism in smut-resistant and susceptible sugarcane genotypes. Transcriptome analysis of smut-infected sugarcane revealed modulation of several genes involved in diverse pathways, including lignin biosynthesis, providing clues for studying the progression of smut disease in sugarcane^5,12^. Another study showed differential expression of 13.5% of genes in the fungal genome at different developmental stages (5 and 200 DAI) of sugarcane–smut infection^14^. Moreover, integrative proteomics and transcriptomics analysis identified 273 and 341 differentially expressed proteins in smut-resistant and susceptible sugarcane genotypes, respectively^13^. These studies reveal significant information about the various transcription-level changes occurring during sugarcane–smut interactions. However, the extent of genome-wide changes in a key post-transcriptional process, alternative splicing (AS), in sugarcane has not yet been reported. This is mainly due to the lack of a sequenced reference genome and the daunting genome-level complexity of sugarcane hybrids with large polyploid genomes (~10 Gbp) and varying numbers of chromosomes^15–17^.

AS generates multiple transcripts (or isoforms) from a single precursor mRNA (pre-mRNA), increasing the diversity and complexity of the transcriptome and proteome^18–22^. AS can lead to truncated proteins and altered levels of protein activity^22,23^. Moreover, AS transcripts with varying sequences result in proteins with altered physical characteristics and molecular functions^20,21^, and it is estimated that 33 to 70%^24^ plant genes undergo AS^25,26^. In addition, AS regulates the level of functional transcripts by a mechanism called regulated unproductive splicing and translation (RUST)^27^. Splicing by the spliceosome complex composed of small nuclear riboproteins (snRNPs) occurs at exon-intron splice sites, usually GT-AG, which play a major role in forming alternative transcripts^22^. Differential splicing of multiexon genes results in four major types of AS: exon skipping (ES), intron retention (IR), alternative donor (AD), and alternative acceptor (AA) types^20,26^.

Several studies have shown that AS in plant genes can be modulated in response to biotic and abiotic stresses^22,23,27^. For example, the *RPS4* disease resistance gene in *Arabidopsis thaliana*^28^ and the *DREB* transcription factor gene in wheat (*Triticum aestivum* L.)^29^ are alternatively spliced in response to biotic stress leading to various disease-induced isoforms. AS is also modulated during plant growth and development, photosynthesis, metabolic pathways, circadian clock function, and flowering^22,26^.

The advent of next-generation sequencing has enabled genome-wide RNA-sequencing studies to examine AS in several, primarily diploid, plants including *Arabidopsis thaliana*^27^, *Brachypodium distachyon*^26^, *Zea mays*^30^, and *Physcomitrella patens*^19^. AS events are often conserved among plant species. For instance, about 58% of AS events are conserved between rice (*Oryza sativa*) and *Arabidopsis thaliana*^22^. Also, AS events in an Arabidopsis splicing regulator gene (*SCL33*) are conserved in Brachypodium and other grasses^26^. In this study, we used RNA sequencing and a hybrid transcript mapping and assembly approach to decipher genome-wide expression changes at the isoform level and modulation of the AS landscapes following infection with *Sporisorium scitamineum* in a sugarcane hybrid showing intermediate smut resistance.

## Results and Discussions

### mRNA sequencing and *Sorghum bicolor* genome-based isoform calling

In the absence of a high-quality reference genome sequence for sugarcane, we leveraged the well-annotated, high-quality reference genome of the related grass *Sorghum bicolor* to establish AS events and isoform expression in sugarcane. During manuscript preparation, a draft monoploid sugarcane genome and *Saccharum spontaneum* genome sequences were released^31,32^. Unfortunately, neither of these published genomes included annotations for alternatively spliced transcripts. Moreover, the draft genomes also used *Sorghum bicolor* reference genome alignments as well as Trinity-based *de novo* transcript assemblies to annotate protein-coding genes. *Sorghum bicolor* and sugarcane share common ancestry, with extensive, genome-wide collinearity (80% with *S. spontaneum* L.) and few chromosomal rearrangements^31–34^. Comparative analysis of *S. bicolor* and sugarcane genome organization indicates that the diploid *S. bicolor* genome is a worthwhile resource for studying the highly complex polyploid sugarcane genome^34^.

**Figure 1:**
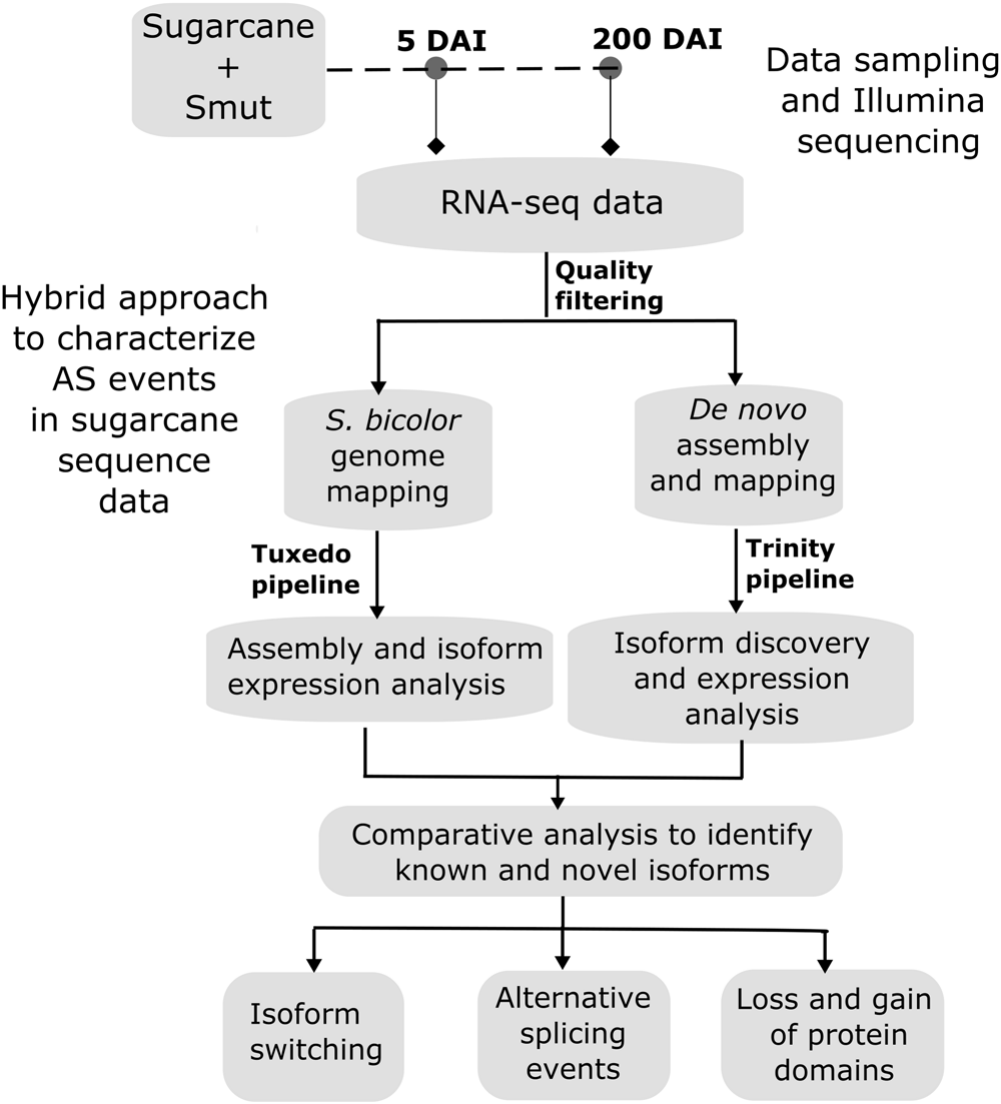
Experimental design and data analysis flowchart. Samples were collected at 5 and 200 DAI and subjected to RNA-seq analysis using three biological replicates for each sample. Twelve libraries were sequenced representing control and stress conditions at the two time points. Data were analyzed using a hybrid approach comprising reference genome (*Sorghum bicolor*)- and *de novo* (Trinity)-based mapping and assembly to identify alternative transcripts and isoform-level regulation.

Twelve samples representing control and smut-infected sugarcane at two stages of infection, i.e., early (5 DAI; before whip emergence) and late (200 DAI; after whip emergence), with three biological replicates each^5^ were subjected to paired-end Illumina HiScanSQ RNA-sequencing (Fig. 1). This produced 112 million raw reads (101 bp) and unambiguously aligned 107 million clean reads (Table 1) to the *Sorghum bicolor* (v3.1 release) genome^35^. The seemingly low overall alignment rate of less than 31% (Table 1) is due to the complex aneuploidy, heterozygous, and interspecific (hybrid) genome of sugarcane, when compared to the *Sorghum* genome. Genome-aligned sequence reads were further processed using Cufflinks, Cuffmerge, Cuffcompare, and Cuffdiff^36^ for discovery of reference and novel isoforms, and isoform-level differential expression analysis.

**Table 1:** Sequence read statistics for all RNA-seq libraries.

Sample	Replicates	Total reads*	Total mapped reads^a^	Total uniquely mapped reads	Multiple mapped reads	Total reads mapped (%)
5 DAI - Control	3	27,783,727 (26,881,845)	16,590,034	12,051,719	4,538,315	30.54
5 DAI - Infected	3	28,179,752 (27,266,828)	16,785,042	12,133,033	4,652,009	30.77
200 DAI - Control	3	28,127,128 (26,228,856)	11,459,114	10,187,726	1,271,388	21.84
200 DAI - Infected	3	28,507,485 (26,628,131)	13,302,600	8,138,565	5,164,035	24.97

*Paired-end RNA-seq reads; values in parentheses indicate cleaned, high-quality sequence reads.

^a^RNA-seq reads mapped to the *Sorghum bicolor* (v3.1 release) genome.

**Figure 2:**
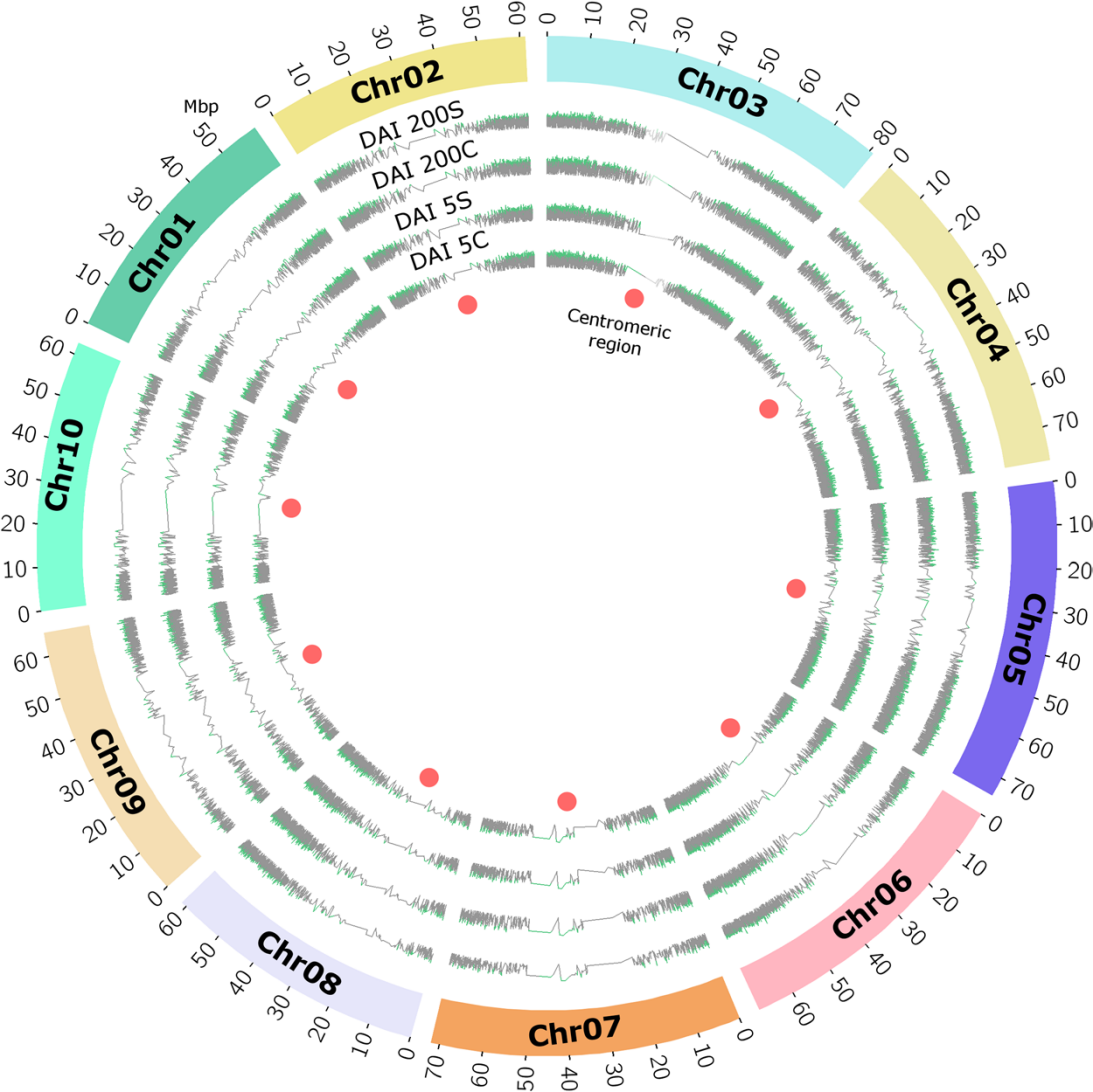
Global distribution of RNA-seq reads along *Sorghum bicolor* chromosomes. RNA-seq read density (log_10_ of absolute read count per gene) of the four sugarcane samples (5 DAI control, 5 DAI stress, 200 DAI control, and 200 DAI stress) across the 10 *Sorghum bicolor* chromosomes. Red dots indicate centromeric regions.

Expression analysis of samples collected at 200 DAI revealed that ~16,000 genes whose homologs are spread across all *Sorghum bicolor* chromosomes had transcriptional activity with log_10_ expression level > 1. To determine gene expression at the chromosome level, we plotted a Circos map of read density along the *Sorghum bicolor* chromosomes (Fig. 2). We observed a uniform distribution of sequence reads along the chromosomes, suggesting no systematic biases (Fig. 2). As expected, heterochromatic regions such as the centromeres showed little to no transcriptional activity. The *Sorghum bicolor* reference-based analysis identified ~52,567 sugarcane transcripts, of which ~4820 were novel isoforms.

### Trinity-based *de novo* isoform calling and annotation

To complement the alignments from *Sorghum bicolor* genome mapping, and to discover novel isoforms, we performed a *de novo* isoform assembly without any predefined genome annotations. We pooled sequence reads from all samples and performed *de novo* transcriptome assembly using Trinity^37^ to construct a consolidated reference transcriptome. Along with reporting assembled transcripts, Trinity resolves alternative isoforms of genes better than other transcriptome assembly tools^37^. Trinity assembled sequence reads into 322,205 transcripts constituting 212,023 unigenes. Among these unigenes, 36,925 had alternatively spliced isoforms (Supplementary Table S1). All unigenes and transcripts were annotated using public protein databases (see Materials and methods) and BLASTX^38^ with e-value cut-off < 1e-05. Priority for annotation was given to *Sorghum bicolor* protein databases^35^ followed by NCBI nr/nt^39^ and UniProtKB^40^ plant protein databases. Among 322,205 transcripts, 142,241 transcripts have shown significant similarity (e-value cut-off < 1e-05) to 28,613 (~60%) of sorghum proteins. Trinity-based *de novo* assembly with annotation using public protein databases allowed us to identify a substantial number of alternatively spliced transcripts.

### Isoform-level expression changes in sugarcane–smut interactions

The *Sorghum bicolor* genome comprises 47,205 transcripts and 34,211 genes^35^. Of the total transcripts assembled by Cufflinks^36^, 15,379 (13,848 known and 1,531 novel) isoforms were expressed at FPKM ≥ 2 at 200 DAI. Similarly, 16,039 (14,423 known and 1,616 novel) isoforms were expressed at FPKM ≥ 2 at 5 DAI. Among these expressed transcripts, 394 and 324 transcripts at 5 and 200 DAI, respectively, did not have any biological annotation in *Sorghum bicolor* databases. The lower number of transcripts during later infection (200 DAI) compared to early infection (5 DAI) could be due to the low mapping rates of the 200 DAI samples (Table 1).

**Figure 3:**
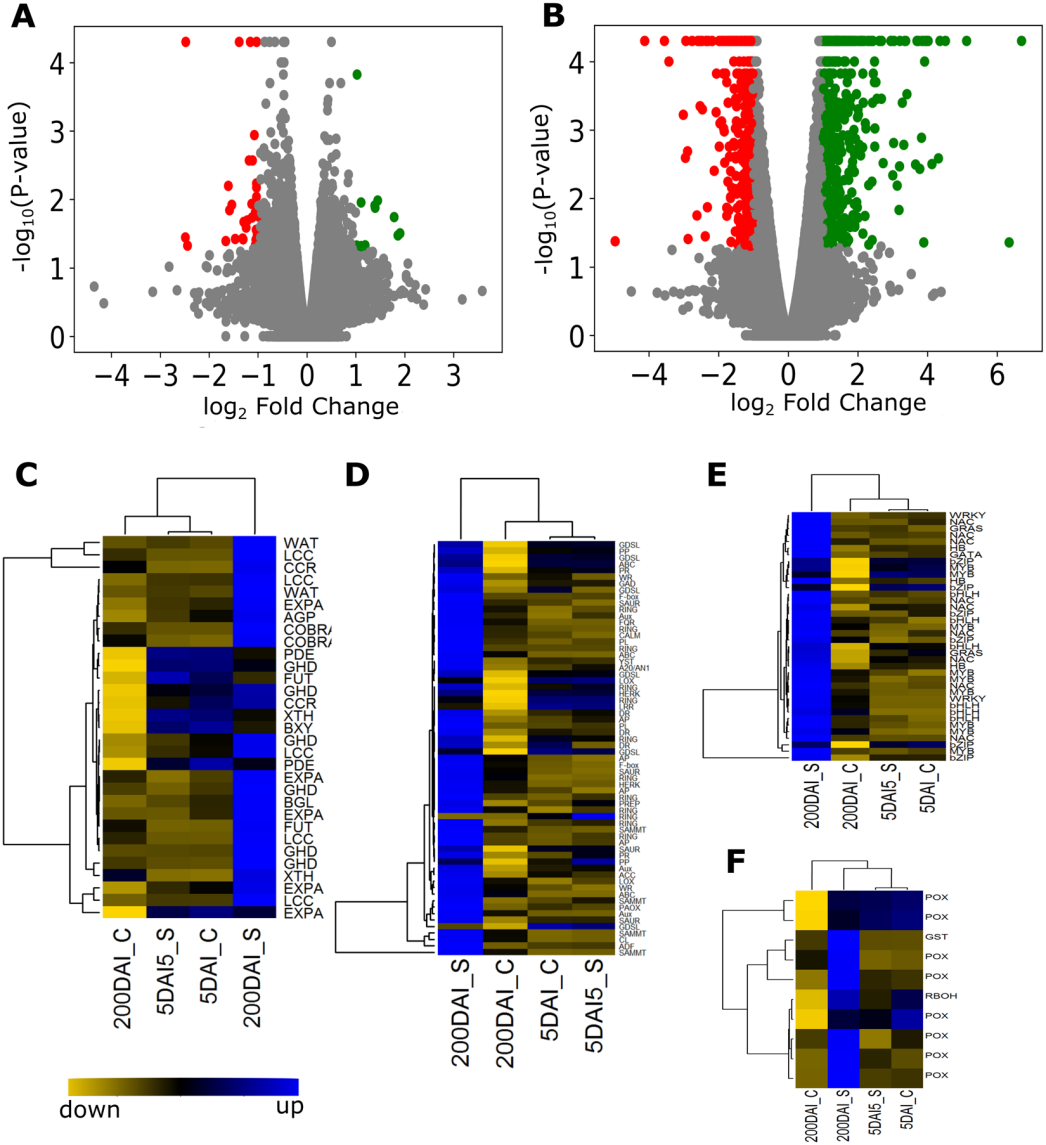
Isoform-level expression analysis in 5 and 200 DAI samples. (**A**,**B**) Volcano plots of differentially expressed isoforms at 5 and 200 DAI, respectively. Green and red dots indicate isoforms significantly upregulated (log_2_ ≥ 1 and P < 0.05) and downregulated (log_2_ ≤ -1 and P < 0.05), respectively, during smut infection. Hierarchical clustering and isoform-level expression dynamics of putative genes associated with (**C**) cell wall modification, (**D**) defense signaling, (**E**) transcription factors, and (**F**) ROS scavenging are represented by heatmaps (blue: upregulated isoforms; yellow: downregulated isoforms). List of the isoforms used in heatmap analysis are provided in Supplementary Data Set 2.

Differential expression analysis of the isoforms was performed using Cuffdiff^36^ to identify changes in AS under control and infected conditions. Low-abundance transcripts with FPKM < 2 were filtered out to preclude transcripts potentially resulting from incorrectly assembled transcripts or sequencing artifacts. We considered isoforms with log_2_ fold change ≥ 1 (2^1^ absolute fold change) at P < 0.05 across control and infected conditions significantly differentially expressed in response to smut infection. Despite greater mapping rates, only 41 transcripts were differentially expressed at 5 DAI, while 855 isoforms were differentially regulated at 200 DAI (Fig. 3A,B; Supplementary Data Set 1). Among them, 530 and 11 isoforms were upregulated, and 325 and 30 isoforms were downregulated in response to smut infection at 200 and 5 DAI, respectively. The greater number of differentially expressed isoforms at 200 DAI compared with 5 DAI might result from enhanced plant stress and defense responses, related to the peak period of whip growth in sugarcane^5^. The 530 upregulated isoforms corresponded to 84 genes that are alternatively spliced or have more than one isoform.

Of the 855 differentially regulated isoforms at 200 DAI, 676 isoforms were supported by the Trinity-assembled transcripts (Supplementary Data Set 1). To be conservative and rigorous in further AS analysis, we only explored differentially expressed isoforms identified by both Trinity and *S. bicolor*-based assembly.

### Sugarcane genome-wide AS landscape affected by smut infection

Biotic and abiotic stresses modulate plant AS landscapes^19,26,30^. We determined AS events in sugarcane under control and smut-stress conditions based on *S. bicolor* mapped data. Using the *S. bicolor* annotation and spliced alignments, we categorized AS events into IR, ES, AD, AA, and other complex events.

**Figure 4:**
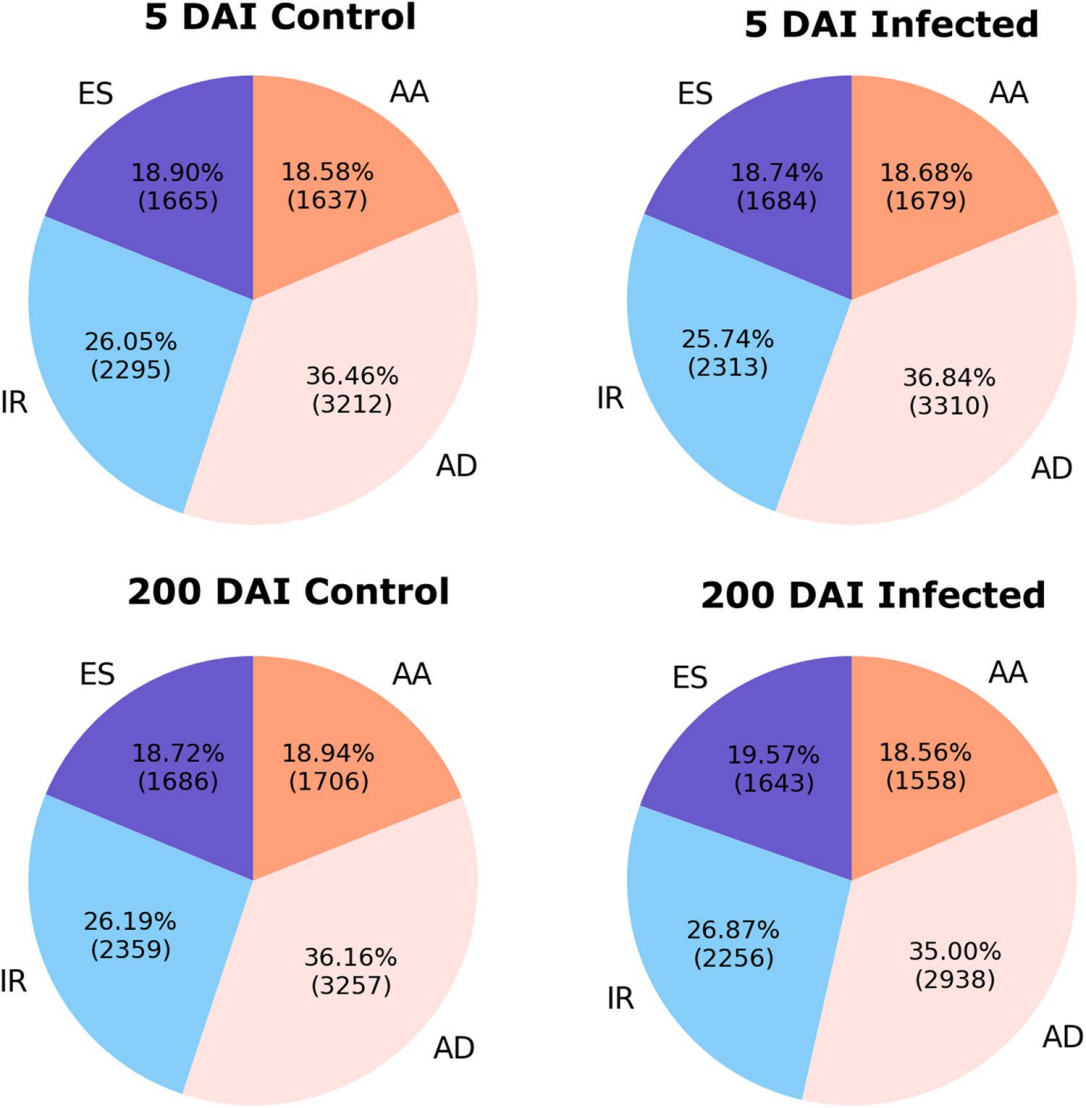
Frequency of AS events identified in RNA-seq data. Distribution of intron retention (IR), exon skipping (ES), alternative donor (AD), and alternative acceptor (AA) AS events under 5 and 200 DAI control (C) and infected (I) conditions.

We found 11,490, 10,699, 11,248, and 11,406 AS events in 200 DAI control, 200 DAI stress, 5 DAI control, and 5 DAI stress conditions, respectively. The distribution of individual IR, ES, AA, and AD events is shown in Fig. 4. AD and AA represented ~50% of the AS events followed by IR (~26%) and ES (~19%) (Fig. 4). We did not see any significant changes in the AS landscape between control and stress conditions, but the proportion of ES (19.57% vs 18.72%) and IR (26.87% vs 26.19%) events was higher under stress conditions than in controls at 200 DAI (Fig. 4).

Among the four major types of AS event, AD events appear to be predominant, while AA and ES were the least common at both 5 and 200 DAI (Fig. 4). The predominance of AD events contrasts with other plant AS landscapes where IR predominates^26,41^. This might be a unique feature of sugarcane, reflecting the complex genomic landscape of this polypoid and aneuploid species. Alternatively, the presence of multiple homeologs with polymorphisms could also result in an overestimation of AD or AA transcripts. Given the lack of a high-quality chromosome-level reference genome and annotation for the hybrid sugarcane, we interpret these results cautiously, and the possible scenarios need to be evaluated in the future.

### Functional enrichment analysis of the alternatively spliced genes

**Figure 5:**
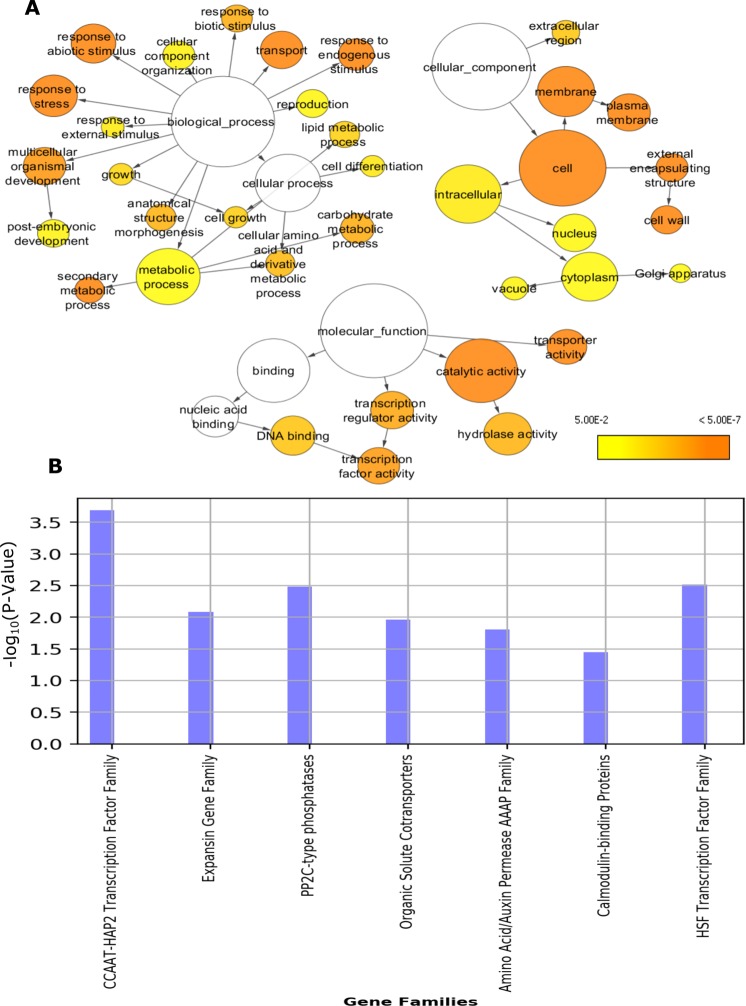
GO and gene family (GenFam) enrichment analysis of induced isoforms in response to smut infection at 200 DAI. (**A**) Enriched GO terms visualized using Cytoscape (see Materials and Methods). GO term interaction networks based on ancestor–child relationships for ‘biological process,’ ‘molecular function,’ and ‘cellular component’ categories. Node size indicates the number of genes in that GO term, and the color scale corresponds to Benjamini-Hochberg P-values. (**B**) Gene family enrichment analysis of induced isoforms in response to smut infection at 200 DAI using the GenFam tool.

To reveal the biological and molecular functions of the 855 isoforms (P < 0.05) that were differentially expressed in response to smut infection at 200 DAI, we performed functional GO and gene family (GenFam^42^; enrichment analyses. We characterized the enriched GO categories (Fisher exact test and FDR < 0.05) and visualized these as interaction networks based on parent–child relationships for ‘biological process,’ ‘molecular functions,’ and ‘cellular components’ (Fig. 5A). To avoid redundant functional analysis by GO categories, we also analyzed the data for gene family enrichment using GenFam. As expected, genes regulating cell wall biosynthesis and/or modifications, transcription factors, and biotic stress responses were highly enriched (Fig. 5B). Enriched GenFam and GO categories contained genes with alternatively spliced isoforms belonging to various functional categories such as cell wall fortification, defense signaling, and transcription factors.

GO terms enriched under the ‘biological process’ category were ‘response to stress’ (GO:0006950), ‘response to biotic stimulus’ (GO:0009607), and ‘transport’ (GO:0006810), while those enriched under ‘molecular functions’ were ‘transcription factor activity’ (GO:0003700), ‘hydrolase activity’ (GO:0016787), and ‘transporter activity’ (GO:0005215). Similarly, in the ‘cellular component’ category, cell wall related terms such as ‘cell wall’ (GO:0005618) and ‘plasma membrane’ (GO:0005886) were highly enriched (Fig. 5A). Comprehensive analysis of GO terms enriched under ‘molecular function,’ ‘biological process,’ and ‘cellular component’ were comparable to each other. Complementing the GO analysis, GenFam allowed identification of overrepresented gene families in biological processes such as cell wall modifications (expansins), transcription factors (HSF), transport (auxin permease), and defense signaling (calmodulin-binding and phosphatases genes) among the alternatively spliced genes (Fig. 5B).

### Alternatively spliced genes perturbed during smut infection

**Figure 6:**
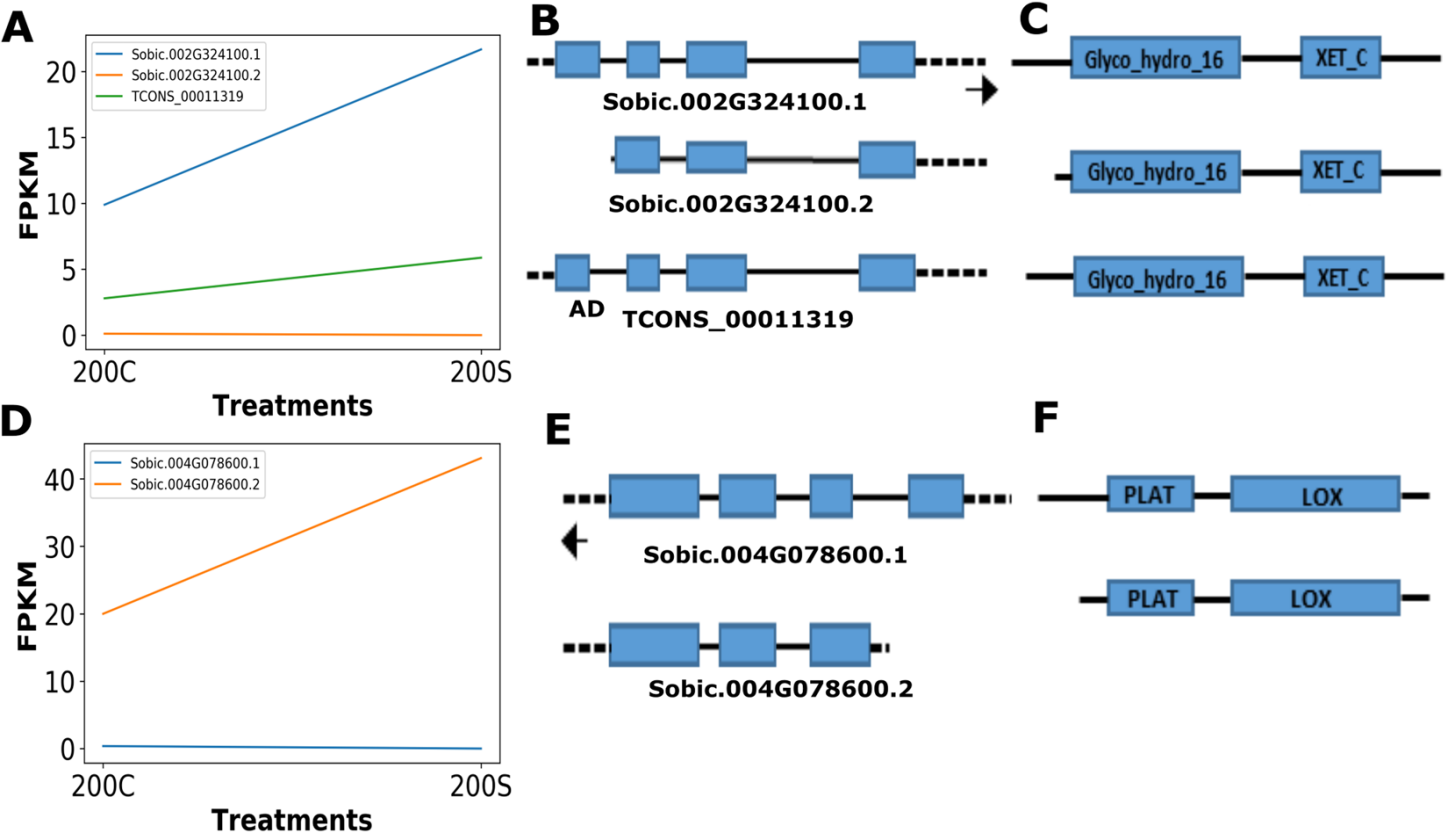
Gene isoform switching at 200 DAI in response to smut infection. Isoform expression changes in genes encoding A) xyloglucan endotransglucosylase/hydrolase (XTH) and D) lipoxygenase (LOX2) (200 C: 200 DAI Control; 200 S: 200 DAI infected). Isoform structures of B) *XTH* and E) *LOX2* with known and novel isoforms, and their corresponding protein structures (C and E). Untranslated and intronic regions are represented by dotted and solid lines, respectively. Left- and right-facing arrows indicate transcript strand orientation.

A significant number of genes underwent AS in response to smut infection at 200 DAI, and some of these were related to defense responses. We categorized the alternatively spliced genes into different functional categories and found that several genes associated with cell wall modifications, transcription factors, ROS scavenging, and defense signaling were alternatively spliced during smut infection (Fig. 3; Supplementary Data Set 1 and 2). Characterization of AS among genes in these categories revealed multiple types of AS ranging from simple to complex isoform switching between control and smut-infected samples (Fig. 6; Supplementary Fig. S1).

**Figure 7:**
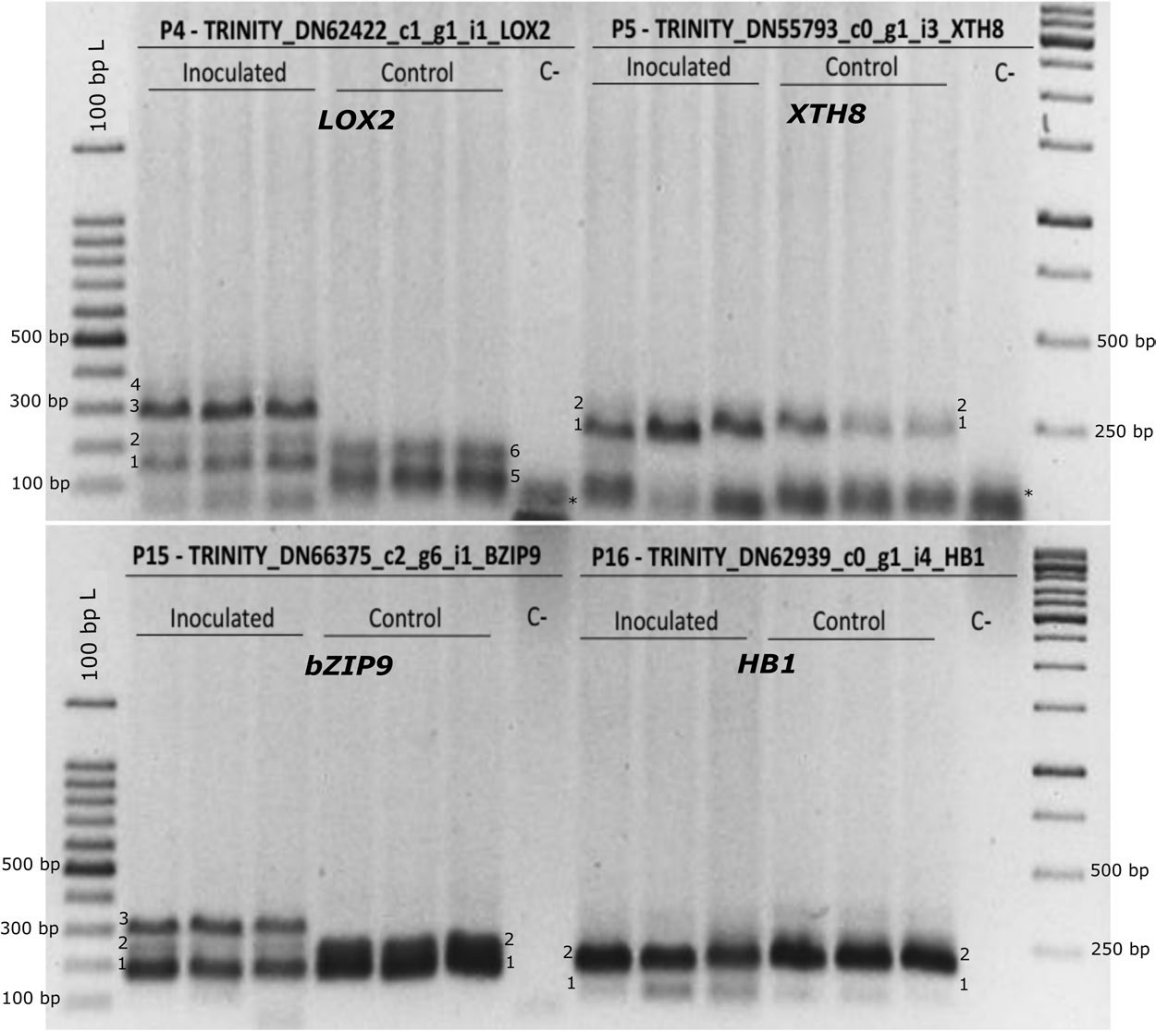
RT-PCR analysis of differentially expressed sugarcane gene isoforms. Alternative splicing in selected sugarcane genes was analyzed using RT-PCR. Molecular size (bp) of the isoforms is shown using a DNA ladder. Putative alternative transcripts/isoforms are labeled numerically. −: negative control/no template; *: primer-dimer. The top and bottom panels were cropped and grouped from different parts of the same gel for clarity. An uncropped image used to prepare this figure is presented in Supplementary Fig. S3.

The cell wall is a primary defense against fungal infection. Several sugarcane genes/isoforms involved in cell wall modification were significantly expressed and alternatively spliced during smut infection (Fig. 3A–C; Supplementary Data Set 1 and 2). For instance, a gene encoding a putative sugarcane xyloglucan endotransglucosylase/hydrolase (*Sobic.002G324100*, *TRINITY_DN55793_c0_g1_i1*, *XTH*, GO:0005618), which could function in cell wall strengthening^43,44^, was differentially regulated and alternatively spliced, with at least three isoforms expressed during smut infection. Increased activity of *XTH* has been linked with the pathogenic defense response by modifying cell wall structure^44,45^. The orthologous gene in sorghum, *Sobic.002G324100*, is annotated as producing two isoforms, with varying 5′ untranslated regions (UTRs). Sugarcane *XTH* has at least three isoforms—two corresponding to sorghum isoforms and a novel isoform predicted by cufflinks. Isoform 1 of *XTH* was the most abundant and could be the primary transcript (Fig. 6A). Isoforms 2 and 3 appeared to have alternative 5′ transcription start sites and exon/intron structures, compared to the primary transcript 1 (Fig. 6B). These *XTH* isoforms also mapped to a locus in the recently published monoploid sugarcane and *Saccharum spontaneum* draft genomes encoding a putative *XTH* gene, *Sh02_t023560* and *Sspon.002C0009350*, respectively. We validated the presence of multiple *XTH* isoforms (numbered 1 and 2) by RT-PCR, and, as predicted, some isoforms were more common in smut-infected samples (Fig. 7). All three isoforms encoded Glyco_hydro_16 and XET_C domains (Fig. 6C), but had alterations in the N-terminal sequence. AS producing changes in the 5′ UTR of *XTH* could also affect transcript stability, translation efficiency and/or subcellular localization^46,47^.

We also identified differential splicing in a defense-related gene cluster (Fig. 3D). A lipoxygenase-encoding gene (*Sobic.004G078600*, *TRINITY_DN62422_c1_g1_i1*, *LOX2*, GO:0009607) was alternatively spliced, with two isoforms differentially expressed during smut infection (Fig. 6D). These isoforms had altered 5′ UTRs with different transcription start sites (Fig. 6E). Isoform 2 appeared to be highly induced in response to smut infection when compared to isoform 1. Both *LOX2* isoforms encoded full-length PLAT and LOX domains with alterations in the N-terminal regions of the proteins (Fig. 6F). As discussed above for *XTH*, alteration of the 5′ UTR of *LOX2* isoforms could affect transcript stability and relative abundance of isoforms encoding functional LOX2. RT-PCR analysis confirmed the presence of up to six *LOX2* isoforms (Fig. 7). Several of these isoforms appeared to be differentially expressed in smut-infected and control samples (Fig. 7). In addition, we found splice variants for additional genes encoding stress-responsive transcription factors such as bZIP and HB by RT-PCR analysis (Fig. 7), providing some empirical evidence for our predictions and demonstrating the utility of the hybrid isoform calling approach (Fig. 1).

In addition to AS, stress induces several types of isoform-level changes in expression or switching^48^. In simple isoform switching, isoforms have varying expression levels but often antagonistic patterns, thus creating little or negligible change in overall gene-level expression (Mandadi and Scholthof, 2015b). In this study, a zinc finger family gene (*Sobic.006G183000)* showed simple isoform switching. Isoform 1 (*Sobic.006G183000.1)* was upregulated under control conditions, whereas isoform 2 (*Sobic.006G183000.2*) was downregulated under stress conditions (Supplementary Fig. S1). In contrast, both *XTH* and *LOX2* genes demonstrated complex isoform switching patterns, with several isoforms showing various expression dynamics (Fig. 6).

To identify the effect of differential splicing on proteins, we determined if AS in multiexonic genes resulted in gain or loss of protein domains. We randomly selected two genes, one encoding tubulin alpha-5 (*Sobic.002G350400*; *TRINITY_DN128043_c0_g1_i1*) and the other encoding a floral homeotic protein (*HUA1*) (*Sobic.001G132200*; *TRINITY_DN69225_c0_g1_i2*) among the genes significantly induced in response to smut infection at 200 DAI. *Tubulin alpha-5* has two known isoforms. *In silico* predictions suggested that isoform 1 has two tubulin domains, comprising the autoregulation signal and tubulin subunits, whereas isoform 2 lacks tubulin subunit (Supplementary Fig. S2B). During infection, only expression of isoform 1 (*Sobic.002G350400.1*) was significantly induced (Supplementary Fig. S2A). Tubulin proteins are major components of microtubules and are crucial for plant cell wall development^49^. AS-mediated changes in the tubulin alpha-5 protein structure could influence the overall levels and/or homeostasis of functional tubulin alpha-5 protein, having biological implications for sugarcane defense responses to smut. Similarly, smut infection induced expression of *HUA1* isoform 1, while levels of isoform 2 were downregulated (Supplementary Fig. S2C). Isoform 1 was predicted to encode a protein with seven zinc finger domains, whereas isoform 2 lacked several zinc finger domains (Supplementary Fig. S2D). Zinc finger domains are required for RNA/DNA binding and play a crucial role in plant development^50^. In a manner similar to tubulin alpha-5, AS-mediated changes in HUA1 protein could have biological implications for sugarcane–smut interactions. Although we made *in silico* predictions and hypotheses regarding how AS could influence protein-level changes, further functional-genetic studies would be needed to test these hypotheses.

## Conclusions

Analysis of AS and isoform-level expression changes in sugarcane has been hampered due to the complex polyploid genome, and lack of a high-quality sequenced reference genome in sugarcane. In this study, we used a hybrid (comparative and *de novo*) transcriptome mapping approach to determine AS patterns and alternatively spliced genes in sugarcane in response to infection with a biotrophic smut fungus. We identified several putative genes with splice variants/isoforms that are differentially expressed during smut infection. The alternatively spliced open reading frames encoded proteins with putative functions in cell wall modification, transcriptional regulation, ROS homeostasis, and defense hormone signaling. Often, AS resulted in truncated or altered protein domains, which could have implications for native protein function, localization and activity. Our study provides the first overview of the genome-wide AS landscape and posttranscriptional gene regulation in the complex sugarcane genome in response to smut infection.

## Methods

### Plant material, RNA extraction, and Illumina sequencing

Sugarcane (*Saccharum* spp.) cultivar ‘RB925345’ showing intermediate smut resistance was inoculated with teliospores of the biotrophic fungus *Sporisorium scitamineum* SSC39 using an artificial wounding protocol^5,14^. Plants were arranged in a randomized block design on greenhouse benches with three replicates of control and fungal treatments. Breaking buds and the culm region (up to 2 cm below culm) were collected and analyzed at an early stage, 5 days after inoculation (DAI), and a later stage after emergence of whips (200 DAI). *Sporisorium scitamineum* infection of sugarcane at 5 DAI was confirmed by rDNA ITS amplicon sequencing using Hs (AACACGGTTGCATCGGTTGGGTC) and Ha (GCTTCTTGCTCATCCTCACCACCAA) primers^5,51^. Total RNA was extracted from frozen tissues at each time point using the protocol described by^14^, and extracted RNA quality was verified using an Agilent 2100 Bioanalyzer (Agilent Technologies, CA, USA). Sequencing libraries were prepared using a TruSeq RNA Sample Prep v2 Low Throughput (LT) kit and as per the Illumina protocol (Illumina, CA, USA). The libraries were subjected to paired-end sequencing on an Illumina HiScanSQ system using Illumina TruSeq SBS reagents.

### Reference-based transcript assembly and isoform calling

RNA-seq libraries from 12 sugarcane samples in response to smut disease (5 and 200 DAI) were filtered using in-house Python scripts to obtain high-quality reads that were mapped to the reference genome of *Sorghum bicolor* (v3.1 release) using the TopHat2 v2.1.1^52^ spliced aligner using Bowtie2^53^ alignment engine. Aligned *Sorghum bicolor* BAM files were further processed by Cufflinks v2.2.1 to assemble the aligned sequence reads into transcripts^36^, guided by the *Sorghum bicolor* annotation to predict novel genes and isoforms. Cufflinks was used to quantify transcript abundances using the fragments per kilobase of exon per million fragments mapped (FPKM) normalization. The Cuffmerge v2.2.1^36^ script was used to create a high-quality merged assembly GTF file and filter out artifactual transfrags for all replicates under each experimental condition. The merged GTF and aligned BAM files among control and experimental conditions were processed by Cuffdiff v2.2.1 to identify significant gene and isoform changes.

### *De novo* assembly, annotation, expression, and functional enrichment analysis

Sequence reads from all 12 RNA-seq libraries were assembled *de novo* using Trinity v2.1.1^37^. The Ada cluster of the Texas A&M University High Performance Research Computing facility (http://hprc.tamu.edu/) with 256 GB memory and 20 core nodes was used to perform sequence assembly. The transcriptome was checked for redundancy using self-BLAST, and exact duplicates were removed. Trinity uses de Brujin graphs and has three core software modules, which first assemble the sequence data into unique contigs (*Inchworm*), cluster the contigs of a given gene and construct a de Brujin graph (*Chrysalis*), and lastly process the clusters and report the full-length alternatively spliced transcripts (*Butterfly*)^54,55^.

The whole transcriptome including alternatively spliced isoforms was annotated using BLASTX^38^ with e-value < 1e-05 against *Sorghum bicolor* proteins, NCBI nr/nt and UniProtKB reference databases. The alignment-based quantification method RSEM^56^ was used to quantify transcript abundance. RSEM uses the Bowtie^53^ aligner to map sequence reads to reference sequences. Differentially expressed transcripts were identified using the edgeR Bioconductor package^57^.

Absolute read counts were calculated using the HTSeq Python framework^58^ with aligned BAM files obtained from TopHat2^58^. Global distribution of mapped RNA-seq reads on each chromosome were visualized using a Circos map^59^. The BiNGO^60^ functional enrichment tool was used for characterizing enriched isoforms with false discovery rate (FDR) < 0.05 for individual gene ontology (GO) terms. Results from BiNGO were visualized as GO networks using Cytoscape^61^. Gene family enrichment analysis was performed using the GenFam^42^ tool with Fisher exact test and Benjamini-Hochberg FDR method.

### Reverse-transcription PCR (RT-PCR) validation of isoforms

Sugarcane ‘RB925345’ single-budded setts were surface disinfected and inoculated with *Sporisorium scitamineum* SSC39 spores (10^6^ teliospores mL^−1^ in saline solution; NaCl_2_ 0.85 M), previously tested for viability (Taniguti *et al*., 2015; Schaker *et al*., 2017). Mock-inoculated plants were prepared with only saline solution. Plants were placed on greenhouse benches in a completely randomized experimental design. After whip emission (120 DAI), meristems of infected and control plants (3 replicates each) were sampled and total RNA extracted using Trizol reagent (Invitrogen). Long cDNAs were obtained using a SMARTer PCR cDNA Synthesis kit (Clontech) and cDNA amplification using an Advantage 2 PCR kit (Clontech), according to the manufacturer’s instructions. cDNAs were diluted 20× and PCR-amplified using Trinity-based primers with a KAPA HiFi HotStart PCR kit (Kapa Biosystems). Reactions contained 1× KAPA HiFi buffer, 0.3 mM KAPA dNTP mix, 0.3 μM each primer, 1 µl diluted cDNA, 0.5 U KAPA HiFi HotStart DNA polymerase, and PCR-grade water to 25 µl. Cycling conditions were as follows: 95 °C for 3 min, 35 cycles of 98 °C for 20 s, 60 °C for 15 s, and 72 °C for 15 s. Results were analyzed in 1.5% agarose gels (0.5× TBE) using 100 bp (Sinapse) and 1 kb (Thermo Scientific) ladders and SYBR Green staining (Invitrogen).

## Supplementary information

Supplementary data

Dataset 1

Dataset 1

## Data Availability

The raw RNA-seq data was deposited in the NCBI SRA database with BioProject accession PRJNA291816^5^. The assembled transcript sequences (excluding transcripts significantly matched to NCBI UniVec database) was deposited at DDBJ/ENA/GenBank database under accession GHKD00000000.
