# Supplementary material for: Genome-wide alternative splicing landscapes modulated by biotrophic sugarcane smut pathogen
Source: Sci Rep. 2019 Jun 20;9:8876. doi: 10.1038/s41598-019-45184-1 (PMC6586842; doi:10.1038/s41598-019-45184-1)
Supplement: Supplementary file 1 — Supplementary data [file 41598_2019_45184_MOESM1_ESM.docx]

Supplementary Data

**Genome-wide alternative splicing landscapes modulated by biotrophic sugarcane smut pathogen**

Renesh Bedre^1^, Sonia Irigoyen^1^, Patricia D.C. Schaker^2, a^, Claudia B. Monteiro-Vitorello^2^, Jorge A. Da Silva^1,3^, and Kranthi K. Mandadi^1,4*^

^1^Texas A&M AgriLife Research & Extension Center, Texas A&M University, Weslaco, TX, USA

^2^Departamento de Genética, Universidade de São Paulo, Escola Superior de Agricultura “Luiz de Queiroz,” Piracicaba, São Paulo, Brazil

^3^Department of Soil & Crop Sciences, Texas A&M University, 2474 TAMU, College Station, Texas 77843

^4^Department of Plant Pathology & Microbiology, Texas A&M University, College Station, TX, USA

^a^Present address: Universidade Tecnológica Federal do Paraná, Toledo, Paraná, Brasil

*corresponding author: kkmandadi@tamu.edu

**
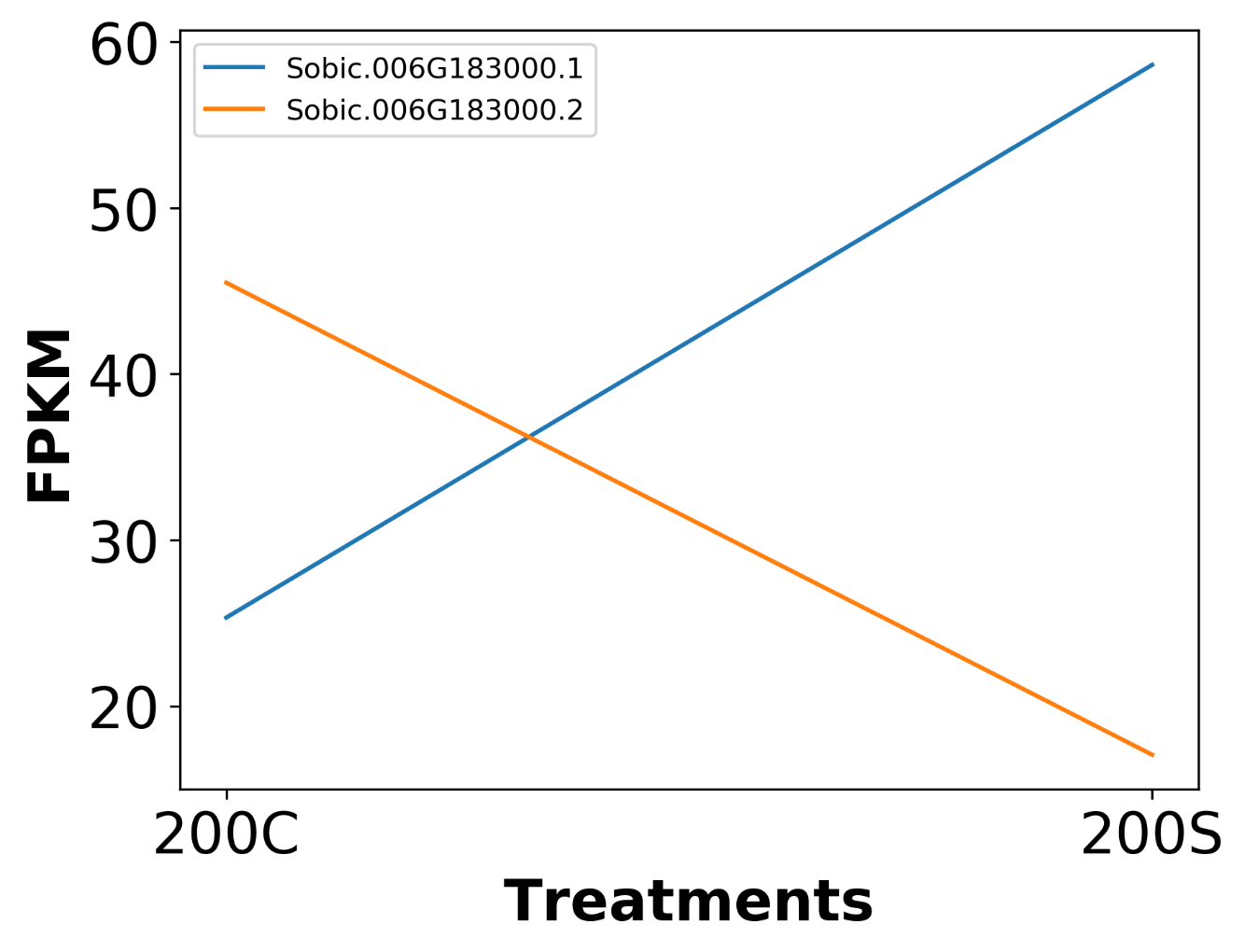
**

**Figure S1.** **Isoform switching of a gene encoding a zinc finger protein**. Changes in isoform expression of a gene encoding a zinc finger protein at 200 DAI in response to smut infection (200C: 200 DAI Control; 200S: 200 DAI infected).

**
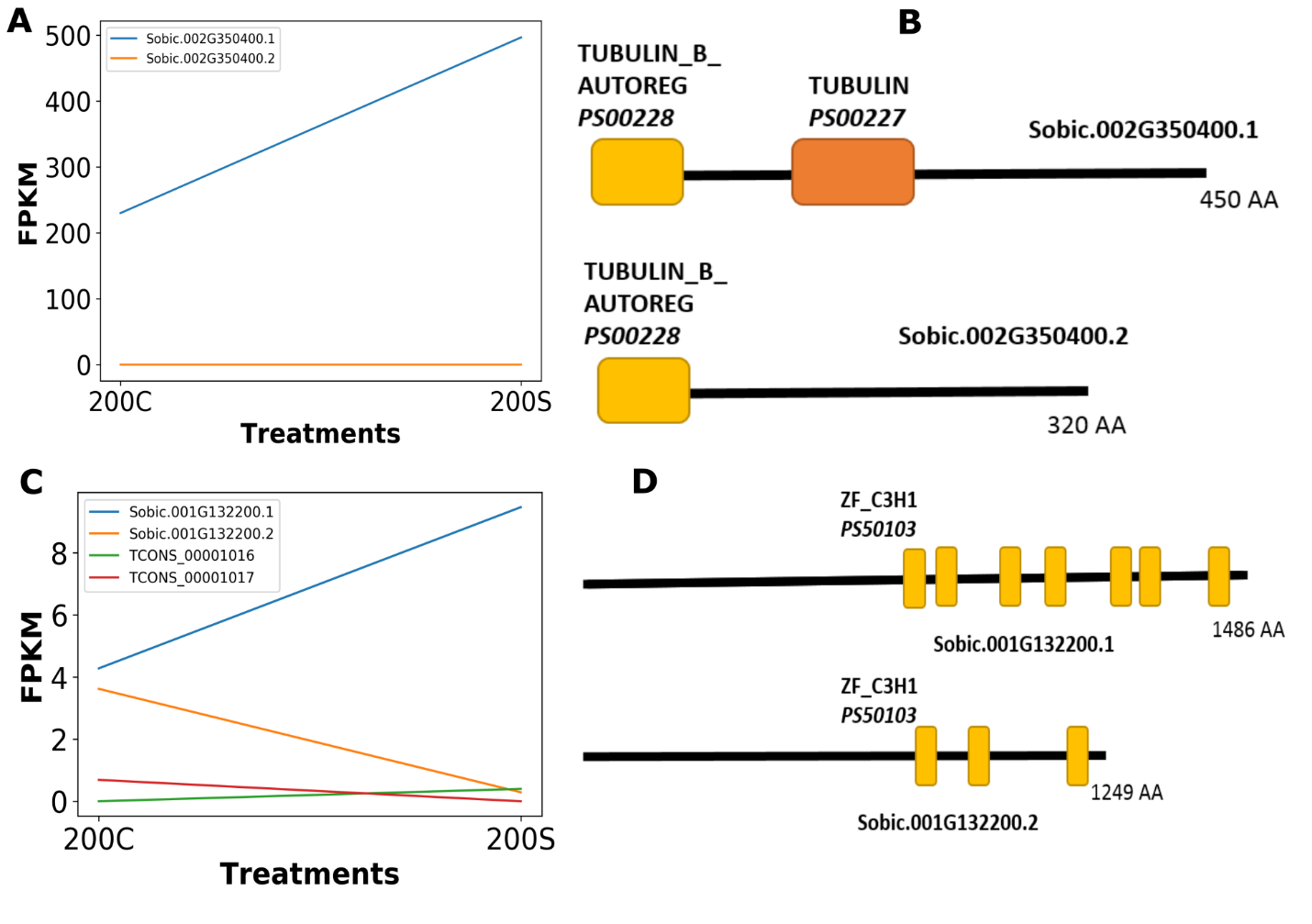
**

**Figure S2. Gain and loss of protein domains induced by alternative splicing.** A) and C) FPKM expression changes under control and stress conditions at 200 DAI (200C: 200 DAI Control; 200S: 200 DAI infected). B) and D) Predicted protein domain dynamics (loss or gain) as a result of AS in the respective isoforms.

**
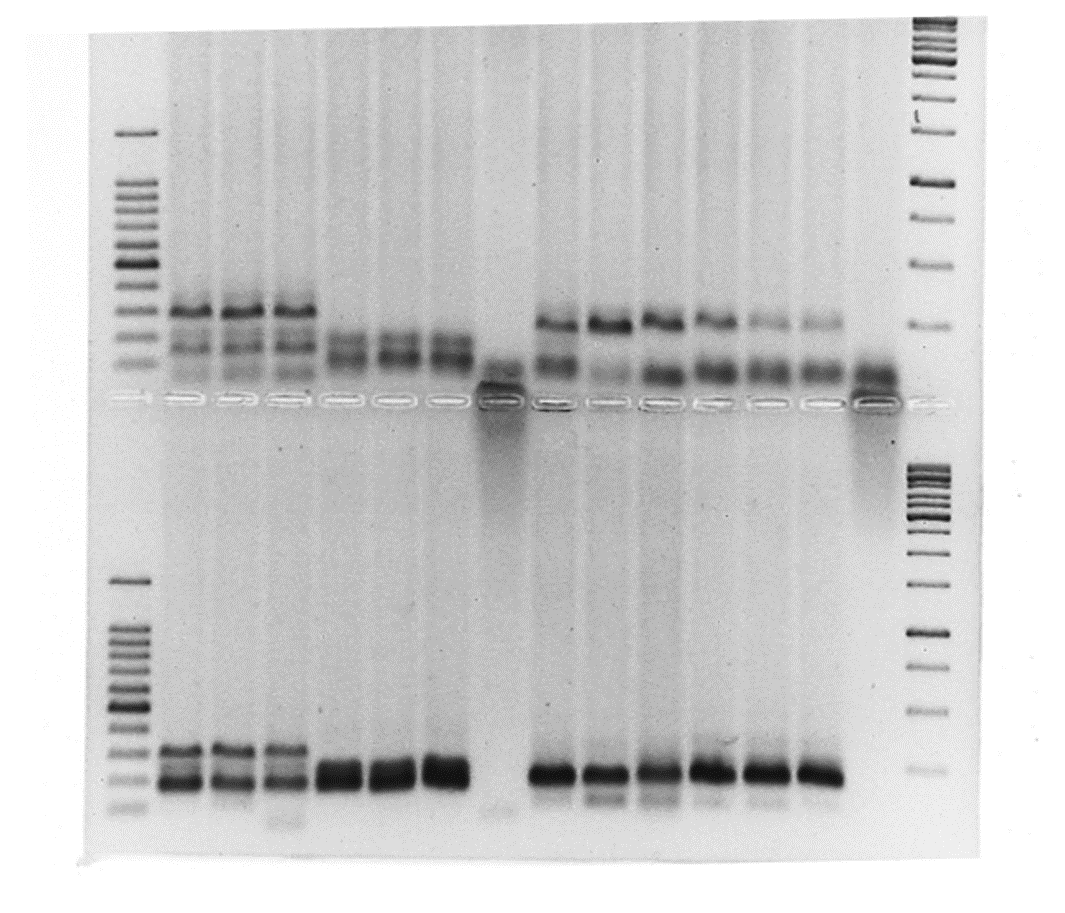
**

**Figure S3.** Uncropped gel image used to prepare Figure 7.

**Table S1**. Trinity assembly statistics

| Parameter | Assembly count |
| --- | --- |
| Total assembled transcripts | 322,205 |
| Total assembled unigenes | 212,023 |
| Total assembled unigenes having isoforms | 36,925 |
| Total assembled bases (bp) | 244,038,570 |
